# Clinical applications of metagenomic next-generation sequencing in the identification of pathogens in periprosthetic joint infections: a retrospective study

**DOI:** 10.1186/s13018-024-04745-5

**Published:** 2024-05-17

**Authors:** Tengfei Shi, Huiyu Chen, Yinhuan Liu, Yexin Wu, Feitai Lin

**Affiliations:** 1 Department of Clinical Laboratory, Fuzhou Second General Hospital, Fuzhou, Fujian China; 2 Department of Clinical Laboratory, Fujian Maternity and Child Health Hospital, Fuzhou, Fujian China; 3 Department of Orthopaedic Surgery, Fuzhou Second General Hospital, Shang Teng Road No. 47 Cang’shan District, Fuzhou, Fujian China

**Keywords:** Periprosthetic joint infection, Metagenomic next-generation sequencing, Microbial culture, *Staphylococcus aureus*, Sinus tract

## Abstract

**Background:**

This study aimed to evaluate the application of metagenomic next-generation sequencing (mNGS) technology to identify pathogens in periprosthetic joint infection (PJI).

**Methods:**

A retrospective analysis was conducted on 65 patients suspected of having PJI between April 2020 and July 2023. The patients were categorized into PJI (46 patients) and non-PJI (19 patients) groups based on the 2018 International Consensus Meeting criteria. Clinical data were collected, and both conventional bacterial culture and mNGS were performed. The diagnostic performance of the two methods was compared and analyzed.

**Results:**

mNGS exhibited a sensitivity of 89.13%, a specificity of 94.74%, a positive predictive value of 97.62%, a negative predictive value of 78.26%, and an overall diagnostic accuracy of 90.77%. Compared to microbial culture, mNGS demonstrated superior diagnostic sensitivity while maintaining similar specificity. A total of 48 pathogens were successfully identified using mNGS, with *Coagulase-negative staphylococci, Streptococci, Staphylococcus aureus,* and *Cutibacterium acnes* being the most common infectious agents. Notably, mNGS was used to identify 17 potential pathogens in 14 culture-negative PJI samples, highlighting its ability to detect rare infectious agents, including *Cutibacterium acnes* (n = 5), *Granulicatella adiacens* (n = 1), *Mycobacterium tuberculosis complex* (n = 1), and *Coxiella burnetii* (n = 1), among others, which are not detectable by routine culture methods. However, mNGS failed to detect the pathogen in 4 culture-positive PJI patients, indicating its limitations. Among the 46 PJI patients, 27 had positive culture and mNGS results. The results of mNGS were concordant with those of culture at the genus level in 6 patients with PJI and at the species level in 18 patients. Furthermore, the present study revealed a significantly greater proportion of *Staphylococcus aureus* in the sinus tract group (45.45%) than in the non-sinus tract group (14.29%), indicating the association of this pathogen with sinus formation in PJI (*P* = 0.03). Additionally, there was no significant difference in the occurrence of polymicrobial infections between the sinus tract group (27.27%) and the non-sinus tract group (33.33%) (*P* = 0.37).

**Conclusions:**

Metagenomic next-generation sequencing can serve as a valuable screening tool in addition to traditional culture methods to improve diagnostic accuracy through optimized culture strategies.

## Introduction

Periprosthetic joint infection (PJI) is a serious complication of joint replacement surgery, with an incidence rate ranging from 2.0 to 2.7% [1]. The complexity of the treatment and management of PJI persists and remains a clinical focus [2, 3]. With the popularization of artificial joint replacement surgery, the incidence of PJI has increased, and PJI poses a significant burden on patients and the health care system [4, 5].

Traditional microbiological culture methods have long been regarded as the “gold standard” for the diagnosis of infection. However, this method has significant limitations, such as a lengthy turnaround time, dependence on viable pathogens, and high standards for culture conditions [6]. Especially in PJI patients requiring urgent intervention, the time required for culture results may lead to delays in clinical treatment and severe adverse outcomes [7, 8]. In recent years, molecular diagnostic techniques have been widely used in the diagnosis of PJI due to their speed, avoidance of antibiotic interference, and minimal sample requirements. However, these techniques, such as multiplex polymerase chain reaction (PCR) and 16S rRNA gene sequencing [9, 10], can only identify a limited number of microorganisms and potentially miss some uncommon pathogens or even fail to detect fungal or polymicrobial infections. Additionally, while matrix-assisted laser desorption ionization time-of-flight mass spectrometry (MALDI-TOF MS) has been widely used in clinical bacterial identification, this method is primarily used for the identification of cultured strains. Although there have been researchers that have attempted to use MALDI-TOF MS to directly assess synovial specimens from blood culture bottles for PJI diagnosis, the sensitivity and specificity of this method are still unable to meet the requirements for PJI diagnosis [11, 12].

To overcome these limitations, metagenomic next-generation sequencing (mNGS) has garnered significant attention. This technology possesses high-throughput detection capabilities, thereby enabling the simultaneous analysis of thousands of DNA fragments and allowing for the comprehensive detection of pathogens such as bacteria, fungi, viruses, and parasites. Diagnostic results are typically available within 48 h [13]. Metagenomic next-generation sequencing has been applied in the detection of pathogens in various infectious diseases, including those affecting the nervous, urinary, and respiratory systems [14–16]. Studies have shown that mNGS exhibits high overall diagnostic sensitivity and specificity in diagnosing PJI, especially in cases where traditional culture methods yield negative results [17–20]. However, despite its substantial theoretical potential, the clinical use of mNGS in the diagnosis of PJI is still relatively limited, and relevant reports are scarce.

Therefore, in this study, we aimed to analyze the clinical characteristics and pathogen distribution patterns of PJIs, comprehensively evaluate the advantages and limitations of mNGS in identifying the pathogen in PJI, and explore the application prospects of mNGS in clinical practice. By increasing the used of mNGS in diagnosing PJI pathogens, we hope to provide clinicians with more accurate, rapid, and comprehensive diagnostic tools to improve the treatment and outcomes of patients with PJI.

## Materials and methods

### Study design

A retrospective analysis was conducted using consecutive patients suspected of having PJI who underwent revision surgery at the Fuzhou Second General Hospital between April 2020 and July 2023. For each patient, demographic characteristics, medical history, clinical signs, laboratory findings, and outcomes from microbial culture and mNGS testing of synovial fluid and periprosthetic tissues were recorded. Ethical approval for this study was granted by the Ethics Committee of the Fuzhou Second General Hospital (Ethical Approval Number: 2021049).

### Inclusion and exclusion criteria

Patients were categorized into PJI and non-PJI groups based on the diagnostic criteria established by the 2018 International Consensus Meeting (ICM) [21]. The inclusion criteria were as follows: (1) a history, clinical signs, or imaging findings suggestive of PJI; (2) provided sufficient samples for microbial culture and mNGS; and (3) complete medical records. The exclusion criteria were: (1) incomplete clinical and laboratory data; (2) insufficient synovial fluid sample volume (< 2 mL); and (3) mNGS results indicating severe contamination of the sample during collection, transportation, or processing. All the results were reviewed by at least two experienced clinicians and an expert laboratory microbiologist to distinguish infection from colonization and contamination.

### Sample collection

During surgery, a strict sterile technique was used to collect synovial fluid and tissue samples from eligible patients. A total of 3 mL of synovial fluid was used for microbial culture, while 0.1–0.5 mL was used to determine the white blood cell (WBC) count and percentage of polymorphonuclear neutrophils (PMNs). The remaining synovial fluid was preserved in sterile, nuclease-free, and amplification inhibitor-free containers at − 80 °C for mNGS. Periprosthetic tissue from at least five different areas was carefully collected intraoperatively to avoid contamination by necrotic tissue. Multiple tissue samples collected from these various areas were immediately dispensed into two separate containers, ensuring that both were sterile and free of nucleases and amplification inhibitors. One tissue sample was immediately sent to the laboratory for culture, and the other was reserved for subsequent mNGS.

### Culture process

Tissue samples were placed in sterile tubes supplemented with 5 mL of brain–heart infusion broth. Subsequently, the samples were sealed, vortexed for 15 min at 2500 rpm, and transferred to a fully automated fast grinder oscillating (Shanghai Jingxin Industrial Development Co., Ltd., Shanghai, China) at a frequency of 60 Hz for 60–120 s. The grinding time and frequency were adjusted based on the collected tissue type to ensure even mixing. The resulting homogenate was inoculated onto blood agar plates and cultured under both anaerobic and aerobic conditions. The remaining samples were inoculated into BACTEC Peds Plus/F bottles and Bactec Lytic/10/F bottles and incubated in the BD automated culture system BACTEC FX (Becton Dickinson and Company, Sparks, MD). The standard culture duration was seven days; however, in specific cases, particularly when the clinical suspicion of PJI was high despite negative cultures, the duration was extended to 14 days to allow for the growth of slow-growing and challenging-to-culture bacteria. Bacterial strain identification was performed using MALDI-TOF MS (Bruker Daltonik GmbH, Billerica, MA) and Phoenix 100 (Becton Dickinson and Company, Sparks, MD).

### mNGS process

We used an automated NGSmaster workstation for nucleic acid extraction, reverse transcription, nucleic acid fragmentation, end repair, 3ʹ end adenylation, sequencing adapter ligation, and purification to construct sequencing libraries. After library quantification using fluorescence quantitative PCR, shotgun sequencing was conducted using the Illumina NextSeq high-throughput sequencing platform. The expected read count for each library was 20 million single-end 50 bp sequence data. We subjected the obtained library sequence data to bioinformatics analysis, filtered out human genome sequence data (GRCh38.p13), and aligned the remaining sequence data to the microbial reference database, which included NCBI GenBank and internally curated microbial genome data, to identify microbial species and determine their relative abundances. Each round of mNGS included a negative control (NC; plasma-free nucleic acids and a fragmented human genomic DNA mixture) and a positive control (a mixture of inactivated bacteria, fungi, and pseudovirus particles). The reporting criteria for mNGS included the following: (1) sequence data meeting quality control requirements (library concentration > 50 pM, Q20 > 85%, Q30 > 80%); (2) NC species not detected on the same chip, or RPM (sample)/RPM (NC) ≥ 5, which was empirically determined based on previous studies as the threshold for distinguishing true positives from background contamination [22–24].

### Statistical analysis

We conducted a statistical analysis of all the data, including clinical characteristics and pathogen detection results. Continuous variables are presented as the mean ± standard deviation, while categorical variables are expressed as frequencies and percentages. We used appropriate statistical methods, such as unpaired t tests, Mann–Whitney U tests, chi-square tests, and Fisher’s exact tests, to compare the performances of the different methods. All the statistical analyses were conducted using GraphPad Prism 9.5, with the significance level set at 0.05.

## Results

### Demographic characteristics

This study included a total of 69 patients with suspected PJI who underwent mNGS, synovial fluid cytology, and bacterial examination. Based on the inclusion and exclusion criteria, four patients were excluded (2 with incomplete data and 2 due to failed testing), resulting in a final analysis of 65 patients with suspected PJI. Among them, 42 patients had undergone total hip arthroplasty (THA), while 23 patients had undergone total knee arthroplasty (TKA). According to the ICM criteria, 46 patients were diagnosed with PJI, while 19 patients were included in the non-PJI group. There were no significant differences in demographic characteristics (such as age, sex, or joint location) or underlying diseases (such as anemia, hypertension, or diabetes) between the PJI and non-PJI groups. However, significant differences in the serum C-reactive protein (CRP) level, erythrocyte sedimentation rate (ESR), D-dimer level, synovial fluid WBC count, synovial fluid PMN (SF-PMN) percentage, sinus tract, hypoproteinemia, antibiotic usage cost, and length of hospital stay were found between the two groups. (Table 1).

**Table 1:** Demographic and clinical characteristics

Characteristics	PJI group (*n* = 46)	Non-PJI group (*n* = 19)	*P*-value
Mean age, yrs (SD)	68.26 (9.86)	65.11 (9.61)	0.24^a^
Sex, n (%)			0.97^d^
Male	22 (47.83)	9 (47.37)	
Female	24 (52.17)	10 (52.63)	
Affected joint, n (%)			0.16^b^
Knee	19 (41.30)	4 (21.05)	
Hip	27 (58.70)	15 (78.95)	
Antibiotic expenditure, CNY(SD)	11,670.19 (20,733.61)	586.78 (1847.62)	< 0.01^c^
Hospital stay, days (SD)	28.33 (16.14)	15.26 (10.87)	< 0.01^c^
Laboratory findings			
SF-WBC, × 10^6^cells/L (SD)	21,811 (38,593)	1034 (701)	< 0.01^c^
SF-PMN, % (SD)	84.11 (13.94)	46.32 (22.66)	< 0.01^c^
CRP, mg/L (SD)	49.04 (44.11)	26.38 (49.43)	< 0.01^c^
ESR, mm/h (SD)	63.13 (28.91)	22.53 (19.58)	< 0.01^c^
D-dimer, mg/L (SD)	6.67 (13.8)	1.49 (1.42)	< 0.01^c^
Sinus tract, n (%)	11 (23.91)	0 (0.00)	0.03^b^
Underling disease			
Anemia	27 (58.70)	10 (52.63)	0.65^d^
Diabetes	11 (23.91)	3 (15.79)	0.74^b^
Hypertension	16 (34.78)	5 (26.32)	0.51^b^
Condition			
Hypoproteinemia	30 (65.22)	7 (36.84)	0.01^d^

*CNY* Chinese Yuan; *SF-WBC* synovial fluid white blood cell count; *SF-PMN* synovial fluid polymorphonuclear cell; *CRP* C-reactive protein; *ESR* erythrocyte sedimentation rate

^a^Independent Sample Unpaired t test; ^b^Fisher's Exact Test; ^c^Mann-Whitney U Test; ^d^Chi-Square Test

### Microbial culture and mNGS results

**Fig. 1:**
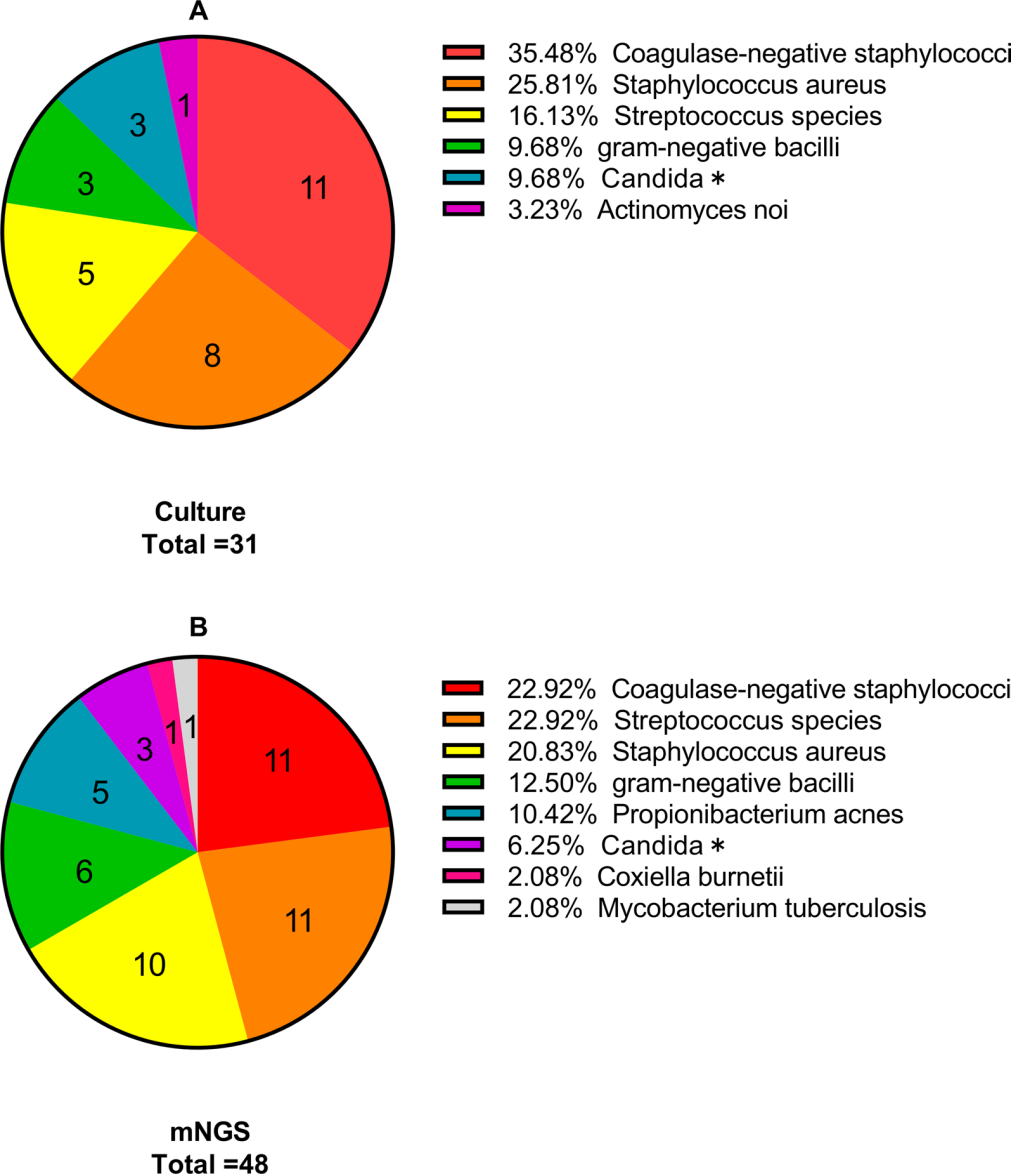
Pie chart showing the proportion of organisms detected by culture (**A**) and mNGS (**B**) in PJIs. *Notes*: The numbers in the pie chart represent the quantity of pathogens; Candida*, including *Candida tropicalis*, *Candida parapsilosis*, and *Candida albicans*. *mNGS* metagenomic next-generation sequencing

Microbiological culture revealed that approximately 67.4% (31/46) of samples in the PJI group were positive for common pathogens, such as *coagulase-negative staphylococci*, *Staphylococcus aureus, Streptococcus species,* and *Candida* species, which were mostly single-species infections. In contrast, mNGS detected the presence of pathogens in 89.13% (41/46) of samples in the PJI group, with 8 cases of multiple infections (cases 3, 9, 15, 21, 22, 28, 41, and 45) and a total of 48 pathogens detected. *Coagulase-negative staphylococci, Streptococcus* species*, Staphylococcus aureus*, and *Cutibacterium acnes* were the 4 most common infectious pathogens. Among the 14 patients with culture-negative prosthetic joint infection (CN-PJI), 80.00% (12/15) received preoperative medication, while only 41.94% (13/31) had culture-positive prosthetic joint infection (CP-PJI). Metagenomic next-generation sequencing detected 11 cases of single-species infections and 3 cases of multispecies infections in CN-PJI, with a total of 17 potential pathogens detected, including pathogens that could not be detected by conventional culture methods, such as *Cutibacterium acnes* (n = 5), *Granulicatella adiacens* (n = 1), *Mycobacterium tuberculosis complex* (n = 1), and *Coxiella burnetii* (n = 1). Notably, 4 cases of CP-PJI, including those caused by *Actinomyces neuii* (n = 1), *Candida parapsilosis* (n = 1), *Staphylococcus epidermidis* (n = 1), and *Staphylococcus xylosus* (n = 1), were missed by mNGS. Additionally, 1 patient was diagnosed with PJI according to the ICM criteria, but neither culture nor mNGS results were able to detect the pathogen. (Table 2 and Fig. 1).

**Table 2:** mNGS and cultured microbiological results in PJI and non-PJI groups

ID	Joint	Administration of antibiotic pre-operatively (Yes, Y/No, N)	Results of mNGS	Results of culture	Sinus tract
*PJI group (n = 46)*					
1	knee	N	*Candida tropicalis*	*Candida tropicalis*	Y
2	Hip	Y	*Staphylococcus epidermidis*	Negative	
3	Hip	Y	*Streptococcus lutetiensis,*	*Streptococcus viridans*	
			*Streptococcus pneumoniae*		
4	Hip	Y	*Coxiella burnetii*	Negative	
5	Hip	N	*Staphylococcus epidermidis*	*Staphylococcus epidermidis*	
6	Hip	Y	*Cutibacterium acnes*	Negative	
7	Hip	N	*Staphylococcus epidermidis*	*Staphylococcus epidermidis*	
8	Knee	N	*Streptococcus sanguis*	*Streptococcus viridans*	
9	Knee	N	*Group B Streptococcus,*	*Group A Streptococcus*	
			*Group A Streptococcus*		
10	Knee	Y	*Group B Streptococcus*	Negative	
11	Hip	N	*Staphylococcus aureus*	*Staphylococcus aureus*	
12	Hip	Y	*Staphylococcus aureus*	Negative	Y
13	Hip	N	*Staphylococcus epidermidis*	*Staphylococcus epidermidis*	Y
14	Hip	N	*Staphylococcus aureus*	*Staphylococcus aureus*	
15	Hip	Y	*Enterococcus faecalis,*	*Klebsiella pneumonia*	Y
			*Klebsiella pneumonia*		
16	Hip	N	*Pseudomonas aeruginosa*	Negative	
17	Hip	Y	*staphylococcus*	Negative	
18	Hip	Y	*Staphylococcus epidermidis*	*Staphylococcus epidermidis*	
19	Hip	N	*Staphylococcus epidermidis*	*Staphylococcus epidermidis*	
20	Hip	N	Negative	*Actinomyces noi*	
21	Hip	N	*Staphylococcus aureus,*	*Staphylococcus epidermidis*	Y
			*Staphylococcus capitis*		
22	Knee	N	*Haemophilus haemolyticus,*	*Haemophilus parahaemolyticus*	
			*Haemophilus influenzae*		
23	Knee	Y	Negative	Negative	
24	Knee	Y	Negative	*Candida parapsilosis*	
25	Hip	N	*Staphylococcus aureus*	*Staphylococcus aureus*	
26	Hip	N	Negative	*Staphylococcus xylose*	Y
27	Hip	N	*Staphylococcus aureus*	*Staphylococcus capitis*	
28	Knee	Y	*Cutibacterium acnes,*	Negative	
			*Escherichia coli*		
29	Hip	N	*Staphylococcus aureus*	*Staphylococcus aureus*	Y
30	Hip	N	*Mycobacterium tuberculosis*	Negative	Y
31	Knee	N	*Acinetobacter baylyi*	Negative	
32	Hip	Y	*Streptococcus sanguis*	*Streptococcus sanguis*	
33	Hip	Y	*Escherichia coli*	*Campylobacter fetus subsp*	
34	Hip	N	*Staphylococcus epidermidis*	*Staphylococcus epidermidis*	
35	Knee	Y	*Candida albicans*	*Candida albicans*	
36	Hip	Y	*Group B Streptococcus*	*Group B Streptococcus*	
37	Knee	Y	*Staphylococcus aureus*	*Staphylococcus aureus*	Y
38	Knee	Y	*Cutibacterium acnes*	Negative	
39	Knee	Y	Negative	*Staphylococcus epidermidis*	
40	Knee	Y	*Staphylococcus aureus*	*Staphylococcus aureus*	Y
41	Knee	Y	*Cutibacterium acnes,*	Negative	
			*Candida parapsilosis*		
42	Knee	Y	*Staphylococcus aureus*	*Staphylococcus aureus*	
43	Knee	Y	*Cutibacterium acnes*	Negative	
44	Hip	N	*Staphylococcus simulans*	*Staphylococcus simulans*	
45	Knee	Y	*Streptococcus anginosus,*	Negative	Y
			*Granulicatella adiacens*		
46	Knee	Y	*Staphylococcus epidermidis*	*Staphylococcus aureus*	
*Non-PJI group (n = 19)*					
1	Knee		*Burkholderia sp*	Negative	
2	Hip		*Negative*	*Cutibacterium acnes*	

The culture and mNGS results of other patients in the non-PJI group were negative

Y, The last column labeled ‘Y’ indicates the presence of a sinus tract

**Fig. 2:**
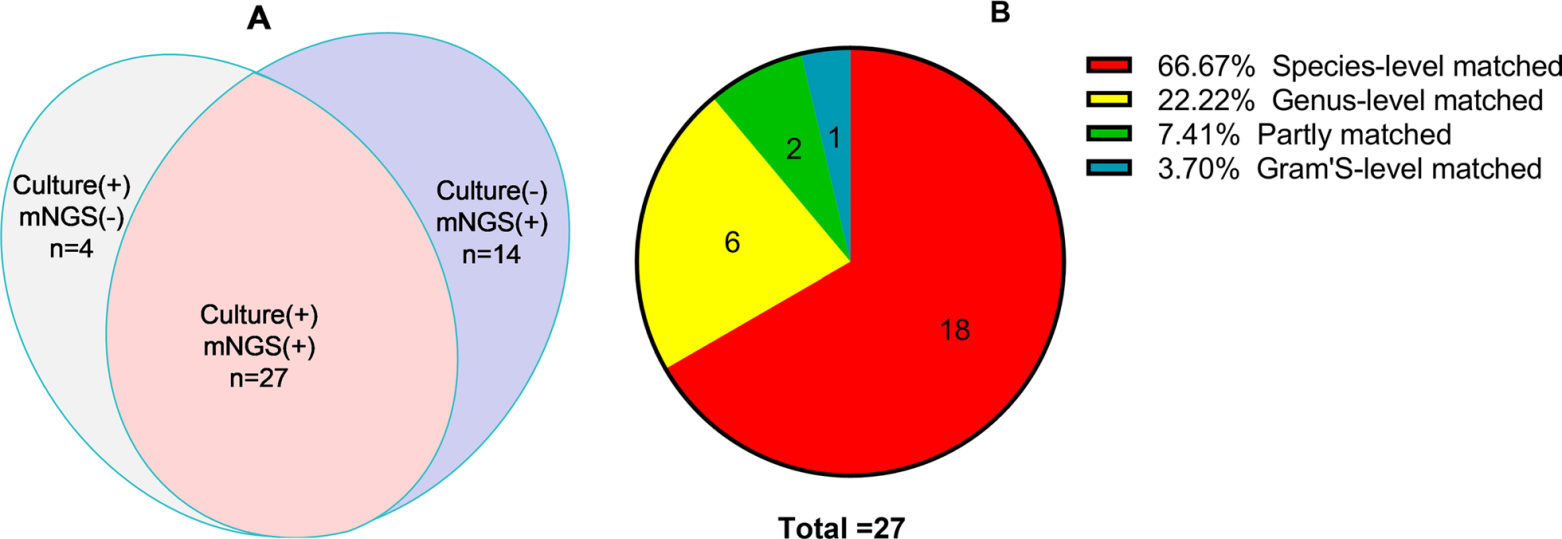
**A** A comparison was made between the number of pathogens detected by mNGS and culture in the PJI group; **B** Consistency between metagenomic next-generation sequencing (mNGS) and culture positivity in prosthetic joint infection (PJI), where n represents the number of patients

Among the 46 PJI patients, 27 had positive culture and mNGS results. The mNGS results of 6 patients with PJI were completely consistent with the culture results at the genus level, those of 18 patients with PJI were completely consistent with the culture results at the species level, and those of 1 patient with PJI were only consistent at the Gram stain level. Additionally, the culture results of 2 patients with CP-PJI were partially consistent with the mNGS results, with mNGS detecting additional potential pathogens, including *Enterococcus faecalis* (n = 1) and *Streptococcus agalactiae* (n = 1) (Fig. 2).

### Diagnostic performance of mNGS for PJI

Based on the combined data, the sensitivity of traditional microbiological culture was 67.39%, with a specificity of 67.39%. The positive predictive value (PPV) was 96.88%, and the negative predictive value (NPV) was 54.55%. The diagnostic accuracy was 75.38%. In contrast, mNGS exhibited superior sensitivity, reaching 89.13%, with a specificity of 94.74%. The PPV of mNGS was 97.62%, and the NPV was 78.26%. The diagnostic accuracy of mNGS was 90.77%. These results indicate that mNGS has greater sensitivity and similar specificity in the diagnosis of PJI (Table 3).

**Table 3:** Comparison of the diagnostic value of mNGS and culture method (%)

Gold standard	Methods	Sensitivity	Specificity	PPV	NPV	Accuracy
ICM	mNGS	89.13	94.74	97.62	78.26	90.77
	Culture	67.39	94.74	96.88	54.55	75.38

*PPV* Positive predictive value; *NPV* Negative predictive value

### Distribution of microbial communities in the sinus tract

**Fig. 3:**
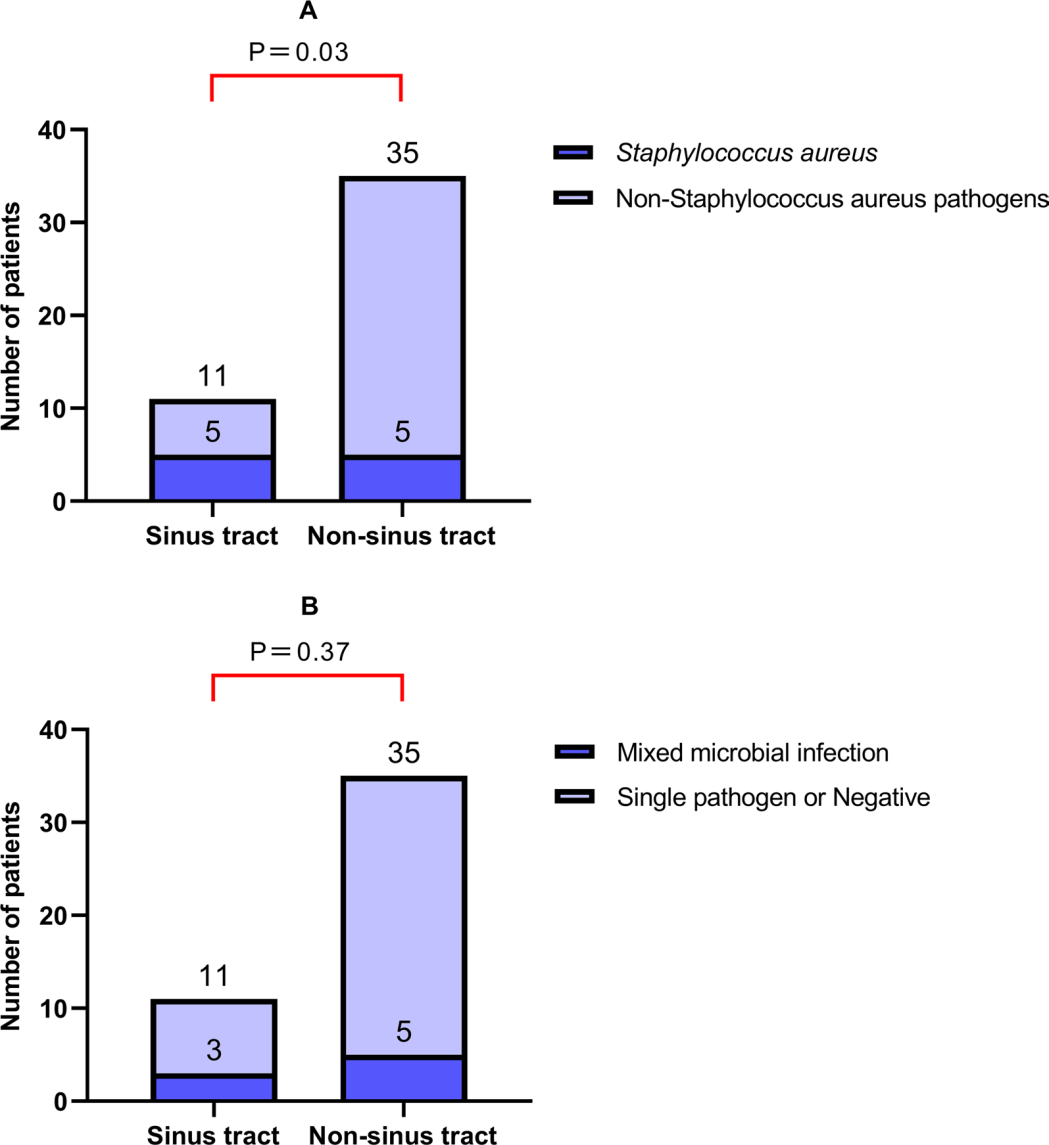
The detection rate of *Staphylococcus aureus* (**A**) and mixed microbial infection (**B**) were compared between the sinus tract group and the non-sinus tract group. *Notes*: **A** The positive rate of *Staphylococcus aureus* was 45.45% (5/11) in the sinus group, whereas it was only 14.29% (5/35) in the non-sinus group. The difference in the detection rate of *Staphylococcus aureus* between the two groups is statistically significant (*P* = 0.03); **B** In the sinus group and the non-sinus group, 27.27% (3/11) and 14.29% (5/35) of patients, respectively, had mixed microbial infections. There was no statistically significant difference between the two groups (*P* = 0.37)

Based on the presence or absence of a sinus tract, 46 patients with PJI were reclassified into two groups: the sinus tract group and the non-sinus tract group. As shown in Table 1, the proportion of microbes detected by mNGS was 90.91% (10/11) in the sinus tract group. Notably, *Staphylococcus aureus* was detected in 5 patients in the sinus tract group, accounting for 45.45% (5/11) of the group. In contrast, the detection rate of *Staphylococcus aureus* was only 14.29% (5/35) in the non-sinus tract group, with a significant difference in the detection rate of *Staphylococcus aureus* between the two groups (*P* = 0.03). Additionally, 27.27% (3/11) of patients in the sinus tract group had multiple infections, compared to 14.29% (5/35) in the non-sinus tract group. No significant difference was observed between the two groups in terms of multiple infections (*P* = 0.37) (Fig. 3).

## Discussion

Periprosthetic joint infection is a severe complication following joint replacement surgery that occurs in between 2.0 and 2.7% of patients and has a significant clinical burden on patients. This infection not only leads to clinical symptoms such as joint swelling and pain but also potentially requires multiple surgeries and joint revision, thereby increasing the risk of patient disability and poor recovery of joint function [25]. The data presented in Table 1 of this study clearly demonstrate that anti-infective treatment in the PJI group significantly increased the length of patients’ hospital stay and antimicrobial drug costs, thus imposing a heavy economic burden on both patients and the health care system.

The diagnostic criteria for PJI proposed by the ICM include laboratory indicators such as ESR, CRP, SF-WBC, and SF-PMN [2]. Although these indicators differed between the PJI and non-PJI groups in our study, their sensitivity and specificity for diagnosing low-grade and chronic PJI remain low [26–29]. This result implies that although these indicators can provide some diagnostic information, they cannot offer direct evidence regarding specific pathogens or accurately distinguish PJI from other joint conditions. Thus, their role in clinical decision-making is relatively limited. Therefore, there is a significant need to explore new diagnostic methods to improve the accuracy of PJI diagnosis.

In this study, mNGS demonstrated a high diagnostic sensitivity of 89.13%, significantly outperforming traditional microbial culture (67.39%). The results of mNGS revealed that *coagulase-negative Staphylococcus*, *Staphylococcus aureus,* and *Streptococcus* species were the most common infectious pathogens of PJI, consistent with findings in other studies [30]. Although microbial culture methods have traditionally been considered the “gold standard” for the diagnosis of PJI, these methods have limitations in the face of CN-PJIs. These limitations may be due to the suppression or elimination of pathogens by antibiotics, leading to negative culture results. The data in Table 2 indicate that 80% of patients with CN-PJI were treated with antibiotics before surgery. Additionally, rare infectious pathogens that cannot grow under standard culture conditions may also result in missed detection. Notably, the advantage of mNGS was particularly pronounced in CN-PJI, where this method was able to detect 14 positive cases among 15 CN-PJI samples, including that caused by pathogens such as *Mycobacterium tuberculosis complex, Coxiella burnetii* [31], and *Granulicatella adiacens*, thus highlighting the potential of mNGS in identifying rare infectious pathogens [32]. These special pathogens are often missed or undetectable by routine culture, even when the culture time is extended to 14 days. The advantage of mNGS lies in its ability to directly detect pathogen nucleic acids without being affected by the use of antibiotics while enabling comprehensive high-throughput detection of all pathogens, thus more accurately reflecting the true infection status of patients. These findings not only deepen our understanding of PJI pathogens but also provide more precise information for clinical treatment.

Although mNGS was able to detect most of the pathogens identified by traditional microbial culture, 4 patients had false-negative results for pathogens in this study. Clinicians conducted a comprehensive assessment of the clinical symptoms and treatment outcomes of these 4 patients, suggesting the possibility of pathogens and the inability to exclude the possibility of infection. These negative results may be caused by various factors. Some Gram-positive cocci and fungi have a thicker microbial wall or a higher lipid content, which makes it difficult to break the wall during nucleic acid extraction, resulting in incomplete release of nucleic acid and reduced DNA yield. Other causes include DNA degradation due to improper sample handling or interference with human DNA. When using mNGS for diagnosis, adhering to standard operating procedures throughout the collection, transportation, and testing processes is crucial to ensure reliability. Considering the substantial cost of mNGS, comprehensively considering its advantages and disadvantages is necessary to select the most appropriate diagnostic method for practical applications. In scenarios where culture results are negative yet PJI remains suspected, mNGS can be utilized as a further diagnostic tool and provide valuable support for clinical decision-making.

Using mNGS technology, we further analyzed the pathogens associated with PJI complicated by the sinus tract. The results of mNGS further revealed a high proportion of *Staphylococcus aureus* infection in the sinus group (45.45%). *Staphylococcus aureus*, which is recognized as a pathogenic bacterium, is prone to invade and spread to surrounding tissues due to its high pathogenicity and virulence, leading to the formation of sinuses [33]. Therefore, a significant difference in the detection rate of *Staphylococcus aureus* between the sinus group and the non-sinus group was expected. Previous studies have indicated that the possibility of detecting multiple microbial infections in sinus samples has increased nearly threefold when using traditional microbial culture methods [34]. With the high sensitivity of mNGS, in this study, we confirmed that the rate of multi-microbial infection in the sinus group was greater than that in the non-sinus group, although there was no statistically significant difference between the two groups. These mixed infections mainly included skin commensal bacteria such as *Enterococcus faecalis*, *Staphylococcus capitis*, and *Streptococcus anginosus*, suggesting that sinuses may provide a pathway for commensal skin bacteria to invade the joint and cause infection. Therefore, in clinical treatment, special attention should be focused on disinfection of the skin around sinus wounds to prevent potential infection risks.

There are several limitations in this study, such as the single-center design, relatively small sample size, and inability to confirm whether the positive results in the non-PJI group represented potential infections or contamination. Therefore, in future studies, we need to further expand the sample size, collaborate with multiple centers, and optimize experimental methods to improve the accuracy of diagnosing PJI.

## Conclusions

In summary, despite some of the challenges and limitations of mNGS [17], such as its high cost, sample contamination issues, inability to detect pathogenic microorganisms in the presence of multiple bacteria, and inability to assess drug sensitivity to guide rational antibiotic selection, its high sensitivity and specificity still support its used as a powerful tool for the diagnosis of PJI. However, traditional microbiological culture methods retain significant value in guiding antibiotic selection. Therefore, although mNGS has high sensitivity, it should not be used as a standalone test. We recommend considering mNGS as a screening tool in addition to traditional culture methods, using results to guide adjustments in culture methods to improve detection rates. In practical applications, we need to comprehensively consider the advantages and disadvantages of this method and combine clinical symptoms, laboratory tests, and culture results to make comprehensive judgments. At the same time, we look forward to more research to further validate and improve the application of mNGS in the diagnosis of PJI.

## Data Availability

The data and materials are available from the medical records department of the Fuzhou Second General Hospital. The datasets used and analyzed during the current study are available from the corresponding author on reasonable request. The original contributions presented in the study are included in the article.

## Abbreviations

mNGS: Metagenomic next-generation sequencing
PJI: Periprosthetic joint infection
MALDI-TOF MS: Matrix-assisted laser desorption ionization time-of-flight mass spectrometry
ICM: International consensus meeting
WBC: White blood cell
PMNs: Polymorphonuclear neutrophils
PCR: Polymerase chain reaction
NC: Negative control
THA: Total hip arthroplasty
TKA: Total knee arthroplasty
CRP: C-reactive protein
ESR: Erythrocyte sedimentation rate
SF-PMN: Synovial fluid polymorphonuclear neutrophil cell
SF-WBC: Synovial fluid white blood cell count
CN-PJI: Culture-negative prosthetic joint infection
CP-PJI: Culture-positive prosthetic joint infection
PPV: Positive predictive value
NPV: Negative predictive value
